# Transcriptome analysis of long non-coding RNAs in *Mycobacterium avium* complex–infected macrophages

**DOI:** 10.3389/fimmu.2024.1374437

**Published:** 2024-04-22

**Authors:** Mitsunori Yoshida, Andrew Taejun Kwon, Xian-Yang Qin, Hajime Nishimura, Shiori Maeda, Yuji Miyamoto, Yasuhiro Yoshida, Yoshihiko Hoshino, Harukazu Suzuki

**Affiliations:** ^1^ Department of Mycobacteriology, National Institute of Infectious Diseases, Higashi-Murayama, Tokyo, Japan; ^2^ Laboratory for Cellular Function Conversion Technology, RIKEN Center for Integrative Medical Sciences, Yokohama, Kanagawa, Japan; ^3^ Department of Immunology and Parasitology, University of Occupational and Environmental Health, Kita-Kyushu, Japan

**Keywords:** non-tuberculous mycobacterium (NTM), bone-marrow-derived macrophage (BMDM), cap analysis of gene expression (CAGE), long non-coding RNA (lncRNA), M1 or M2 macrophage, Ingenuity Pathway Analysis (IPA)

## Abstract

*Mycobacterium avium* complex (MAC) is a non-tuberculous mycobacterium widely distributed in the environment. Even though MAC infection is increasing in older women and immunocompromised patients, to our knowledge there has been no comprehensive analysis of the MAC-infected host-cell transcriptome—and particularly of long non-coding RNAs (lncRNAs). By using *in vitro-*cultured primary mouse bone-marrow-derived macrophages (BMDMs) and Cap analysis of gene expression, we analyzed the transcriptional and kinetic landscape of macrophage genes, with a focus on lncRNAs, during MAC infection. MAC infection of macrophages induced the expression of immune/inflammatory response genes and other genes similar to those involved in M1 macrophage activation, consistent with previous reports, although *Nos2* (M1 activation) and *Arg1* (M2 activation) had distinct expression profiles. We identified 31 upregulated and 30 downregulated lncRNA promoters corresponding respectively to 18 and 26 lncRNAs. Upregulated lncRNAs were clustered into two groups—early and late upregulated—predicted to be associated with immune activation and the immune response to infection, respectively. Furthermore, an Ingenuity Pathway Analysis revealed canonical pathways and upstream transcription regulators associated with differentially expressed lncRNAs. Several differentially expressed lncRNAs reported elsewhere underwent expressional changes upon M1 or M2 preactivation and subsequent MAC infection. Finally, we showed that expressional change of lncRNAs in MAC-infected BMDMs was mediated by toll-like receptor 2, although there may be other mechanisms that sense MAC infection. We identified differentially expressed lncRNAs in MAC-infected BMDMs, revealing diverse features that imply the distinct roles of these lncRNAs in MAC infection and macrophage polarization.

## Introduction

Non-tuberculous mycobacteria (NTMs) include all mycobacteria with the exception of *Mycobacterium tuberculosis* (Mtb) complex and *Mycobacterium leprae*. Unlike Mtb, NTMs are thought to exist in the natural environment, which is the usual source of human infection. In contrast to the decline in pulmonary Mtb infection, the number of patients with pulmonary NTM diseases has been increasing worldwide in recent years (1–5). Although a complex relationship among environmental, mycobacterial, and host factors has been suggested in the pathogenesis of NTM diseases, much of the pathogenesis mechanism remains to be elucidated.

The dominant type of pulmonary infection caused by NTM varies from country to country (6). In Japan, pulmonary infectious disease caused by *Mycobacterium avium* complex (MAC: *Mycobacterium avium* and *Mycobacterium intracellulare*) accounts for about 90% of all pulmonary NTM infections (1, 7). In the past, pulmonary NTM disease was frequently observed in patients with immunodeficiency conditions such as AIDS, carcinomatosis, or silicosis, or in patients with underlying respiratory diseases such as post-tuberculosis syndrome, COPD, or cystic fibrosis that left cavities in the lungs (8). However, in recent years, an increase in pulmonary NTM disease has been observed even in patients whose immunity is considered normal. In particular, pulmonary NTM disease is now frequently diagnosed in postmenopausal middle-aged and elderly females, rather than in males, suggesting deterioration of the host immunity (2).

The first step in the host protecting from NTM infection would be an innate immunity, largely depending upon pattern recognition receptors (PRRs). Among them, toll-like receptor 2 (Tlr2) serves as one of the most important PRR to sense toward an invasion of the external pathogens. Most of the progress about *Mycobacterium* infection to Tlr2 has been made in defensing Mtb. There are several reports Tlr2 senses invading Mtb through the lipoproteins and glycolipids on the cell wall that are also located in NTM, such as MAC.

Knowledge of specific pathways for immune invasion may provide insights into host-pathogen interactions that determine the outcome of infection. Pathogen-induced changes are generally accompanied by remarkable changes in gene expression due to host- and pathogen-mediated reprogramming of the transcriptome during infection (9).

Several studies have shown that RNA sequencing analysis is helpful for understanding the overall host response to NTM infection (10–12), but analyses of the transcriptomes of host macrophages, which are thought to be the first cells to respond upon MAC infection, are limited. Furthermore, no published reports have examined the dynamics of long non-coding RNAs (lncRNAs) in MAC infection, although lncRNAs are known to be involved in diverse biological processes such as cell differentiation, oncogenesis, ontogeny, individual development, and disease, including bacterial infection, through regulation of transcription, translation, and epigenetics (13, 14).

Here, we analyzed the transcriptome of host macrophages by using a *Mycobacterium avium* subsp. *hominissuis* (MAH) strain, TH135, which is highly pathogenic among MAC strains (15), and a highly sensitive RNA sequencing method (CAGE-seq, Cap analysis of gene expression) that we developed (16). We found that MAC infection of macrophages induced the expression of immune/inflammatory-response genes and other genes similar to those involved in M1 macrophage activation, although *Nos2* (involved in M1 activation) and *Arg1* (involved in M2 activation) had distinct expression profiles. Furthermore, we identified the association of host macrophage lncRNAs with MAC infection, about which little had been known until now.

## Materials and methods

### Generation of bone-marrow-derived macrophages

Bone-marrow-derived macrophages (BMDMs) were generated from 8- to 12-week-old BALB/c male wild-type mice (CLEA Japan Inc., Tokyo, Japan) and Tlr2 knockout mice (Oriental Bioservice, Inc., Kyoto, Japan) as described previously, with some modifications (17). In brief, bone marrow cells were harvested from femurs. Cells were cultured in 90 × 15-mm vented petri dishes (Sansei Medical Co. Ltd., Kyoto, Japan) for 10 days at 37°C under 5% CO_2_ in RPMI-1640 medium (Fujifilm Wako Pure Chemicals, Ltd., Osaka, Japan) containing 10% FBS (Thermo Fisher Scientific Inc., Waltham, MA, USA), 40 ng/mL GM-CSF (BioLegend, San Diego, CA, USA), 20 ng/ml M-CSF (BioLegend, San Diego, CA, USA), and 50 μ/mL ampicillin (Sigma-Aldrich, St. Louis, MO, USA) (the RPMI-1640 medium). After 10 days, BMDMs were harvested for subsequent experiments.

### Preparation of BMDMs infected with MAH strain and treated with Tlr2 ligand

BMDMs were plated in the RPMI-1640 medium in six-well plates (BD Falcon, NJ, USA) at 2 × 10^6^ cells per well and kept overnight at 37°C under 5% CO_2_. The BMDMs were then infected with log-phase MAH strain TH-135 and left for 4 h. The medium was replaced with fresh medium to remove extracellular mycobacteria. The cells were harvested at 0, 4, 12, or 24 h post-infection. To prepare BMDMs treated with Tlr2 ligand, 2 × 10^6^ cells were incubated in the RPMI-1640 medium containing the Tlr2 ligand MALP-2 (macrophage-activating lipopeptide-2) (Enzo Life Sciences Inc., Farmingdale, NY, USA) for 0, 4, or 24 h. After the treatments, cells were harvested and lysed with 700 μL of QIAzol (Qiagen, Valencia, CA, USA) and then stored at –80°C for RNA extraction. Total RNA was prepared by using an miRNeasy kit (Qiagen, Valencia, CA, USA). RNA quality was assessed with a Bioanalyzer (Agilent Technologies, Palo Alto, CA, USA) to ensure that the RNA integrity number was over 7.0 and that the ratio of absorbance at 260 nm to that at 280 nm, and that at 260 nm to that at 230 nm, exceeded 1.7. Quadruplicate samples were produced at each time point.

### Transcriptome analysis

Transcriptome analysis of MAC-infected BMDMs was performed by using CAGE. In brief, CAGE libraries were constructed by using the published nAnT-iCAGE (non-amplified non-tagging Illumina CAGE) protocol (16), followed by sequencing. The sequenced reads were processed by using the MOIRAI pipeline (18). After being filtered for ribosomal and low-quality reads, they were mapped to the mouse genome (mm9) by using BWA version 0.5.9 (r16) to calculate gene expression (19). Differential expression analysis was performed with edgeR (version 3.28.0) (20).

### Quantitative reverse transcription – polymerase chain reaction analysis

Total RNA was reverse-transcribed by using Superscript III Reverse Transcriptase (Thermo Fisher Scientific Inc., Waltham, MA, USA) followed by PCR amplification of target genes. Glyceraldehyde-3-phosphate dehydrogenase mRNA was used as a control for data normalization. Changes in gene expression were determined by using the 2^−ΔΔCt^ method.

### Prediction of the role of differentially expressed lncRNAs

To explore the biological interpretation of differentially expressed lncRNAs, we listed protein-coding transcripts with Pearson correlation coefficients of both more than 0.8 and less than -0.8 with each differentially expressed lncRNA by using the time-course transcriptome data. The list underwent Gene Ontology (GO) enrichment analysis. The enriched GO biological process was analyzed by using the web-based ToppCluster tool (https://toppcluster.cchmc.org/) (21). The list (Pearson correlation coefficients of both more than 0.9 and less than -0.9 with each differentially expressed lncRNA) was also used for Ingenuity Pathway Analysis (IPA) (Ingenuity Systems, Mountain View, CA, USA) to predict canonical pathways and upstream transcription regulators associated with differentially expressed lncRNAs. The activation z-score was used as the statistical measure in the IPA analysis to find likely regulating molecules on the basis of the statistically significant pattern match of up- and downregulation, as well as to predict the state of activation (either activated or inhibited) of the putative canonical pathway or upstream regulator (22). An absolute z-score of 2 or more was considered significant.

### Preparation of MAC-infected BMDMs under M1 and M2 macrophage preactivation

BMDMs were plated in the RPMI-1640 medium in six-well plates (BD Falcon, Franklin Lakes, NJ, USA) at 2 × 10^6^ cells per well and incubated overnight at 37°C under 5% CO_2_. This was followed by stimulation with IFNγ (100 units/mL, BD Biosciences, San Jose, CA, USA) or IL-4/IL-13 (100 units/mL each, BD Biosciences) for 24 h at 37°C under 5% CO_2_. Cells were infected with MAH and left for 4 h, then washed to remove extracellular mycobacteria. They were then incubated until 24 h post-infection. At 0 and 24 h post-infection, cells were harvested and the RNA extracted.

### Analysis of *Tlr2*-knocked-out BMDMs infected with MAC

BMDMs were prepared from wild-type and Tlr2 knockout mice, as described above. BMDMs were infected with MAH (with a multiplicity of infection (MOI) of 200) and harvested at 0 and 24 h. This was followed by RNA extraction. Expressional change of representative lncRNAs was measured by RT-qPCR.

## Results

### Transcriptional landscape of MAC*-*infected macrophages

Macrophages are innate immune cells that are the primary targets of *Mycobacterium* infection. By using *in vitro-*cultured primary mouse BMDMs, we designed and characterized the transcriptional and kinetic (0, 4, 12, 24 h) landscape of the macrophages during MAC infection. We decided to use an MOI of 200 for MAC infection after we analyzed the results of a preliminary experiment (**Supplementary Figure S1**) and consulted the literature (11, 23). Total RNA was extracted from harvested cells and subjected to nAnT-iCAGE transcriptomics (16).

To characterize the promoter-level gene expression of MAC-infected macrophages (**Supplementary Table S1**), we extracted differentially expressed gene promoters (>5 log counts per million (cpm) expression, >2-fold change, false discovery rate < 0.01) at each time point (**Supplementary Tables S2** **–** **S4**). At each time point, several hundred genes were up- or downregulated in comparison with their expression at 0 h. To explore the global effect of MAC infection of macrophages, differentially expressed genes underwent GO analysis at each time point (**Supplementary Tables S5** **–** **S7** for upregulated genes and **Supplementary Tables S8**-**S10** for downregulated genes). At 4, 12, and 24 h, identification of the genes upregulated by MAC infection revealed an enrichment of similar GO biological process terms (**Supplementary Tables S5** **–** **S7**). The ontology terms “immune system process” (GO: 0002376), “defense response” (GO: 0006592), and “response to stress” (GO: 0006950) were among the top 20 terms at all time points. Furthermore, the ontology term “immune response” (GO: 006955) was enriched in the top 20 terms at 12 and 24 h and “inflammatory response” (GO: 0006954) at 4 h. The results of the analysis suggested that an immune/inflammatory response, characteristic of M1 macrophage activation, occurred in MAC-infected macrophages.

**Figure 1:**
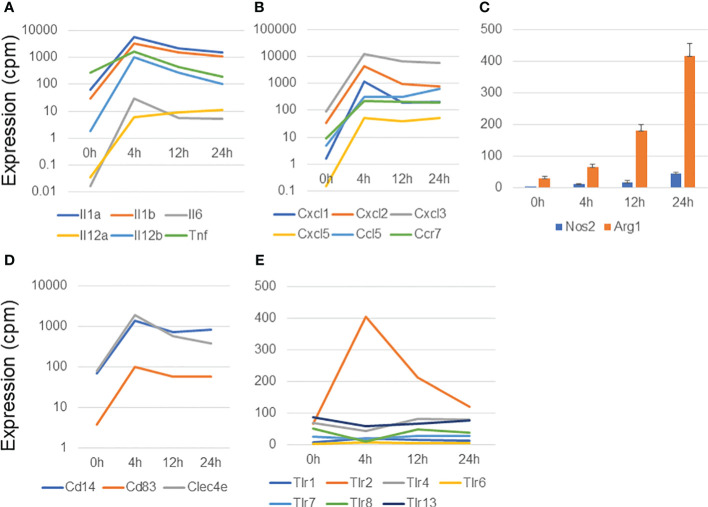
Analysis of genes upregulated in macrophages infected with *Mycobacterium avium* complex. CAGE-seq transcriptome analysis was performed at 0, 4, 12, or 24 h post-infection. Average expression values of quadruplicate data were plotted in each figure. The standard deviation was omitted except for **(C)**. **(A)** Induction of representative cytokine gene expression. **(B)** Induction of representative chemokine gene expression. **(C)** Expressional change of *Nos2* and *Arg1*. **(D)** Induction of membrane protein genes, *Cd14*, *Cd83*, and *Clec4e*. **(E)** Transient induction of *Tlr2* among the *Tlrs*.

We had previously described the gene expression profiles of BMDMs that are associated with IFNγ-stimulated M1 activation (24). Therefore, we examined the characteristics of the genes upregulated in MAC-infected macrophages and compared them with those upregulated in IFNγ-stimulated M1 macrophage activation (**Figure 1**; **Supplementary Figure S2**). We found that the genes encoding inflammation-related cytokines (*Il1a*, *Il1b*, *Il6*, *Il12a*, *IL12b*, and *Tnf*) (**Figure 1A**) and chemokines and their receptor (*Cxcl1*, *Cxcl2*, *Cxcl3*, *Cxcl5*, *Ccl5*, and *Ccr7*) (**Figure 1B**) were dramatically upregulated at 4 h in MAC-infected macrophages. Their expression then gradually declined at 12 and 24 h. Most cytokine and chemokine genes underwent similar upregulation in IFNγ-stimulated M1 activation (**Supplementary Figures S2A, B**).

The production of two key enzymes, Nos2 and Arg1, is induced in M1 and M2 activation, respectively (25, 26). Nos2 plays an essential role in the enhancement of antimicrobial activity, whereas Arg1 competes with Nos2 for the same substrate, L-arginine, and is involved in the cell-wounding response. *Arg1* was predominantly upregulated in MAC-infected macrophages, although the expression of both *Nos2* and *Arg1* gradually increased from 4 to 24 h (**Figure 1C**). In contrast, in IFNγ-stimulated M1 activation, *Nos2* expression was transiently induced at 4 h and then declined at 12 and 24 h, whereas *Arg1* expression was barely detectable (**Supplementary Figures S2C, D**). Similarly, in a previous study of Mtb-infected BMDMs (17), transient *Nos2* upregulation was predominantly observed, whereas *Arg1* was barely expressed (**Supplementary Figures S2E, F**). Because MAC is known to be sensed predominantly by Tlr2 (27), we stimulated BMDMs with MALP2, a Tlr2 agonist, and explored *Nos2* and *Arg1* expression. Interestingly, transient induction of *Nos2* expression was observed in MALP2-stimulated BMDMs (**Supplementary Figure S2G**), whereas *Arg1* expression was gradually increased, similarly to the case in MAC infection (**Supplementary Figure S2H**; **Figure 1C**). We also confirmed no upregulation in the expression of the M2 marker genes encoding galectin 3 (*Lgals3*) and mannose receptor, C type 1 (*Mrc1*) (**Supplementary Figure S2I**).

Finally, we found upregulation of several membrane protein in MAC-infected macrophages. Of those, *Cd14*, *Cd83* and *Clec4e* were dramatically upregulated in MAC infection (**Figure 1D**). Toll-like receptors (Tlrs) are protective immune proteins that detect pathogen-associated molecules. We observed transient *Tlr2* induction among *Tlr* family at 4 h in MAC-infected macrophages (**Figure 1E**), which was consistent with previous report (27).

Taken together, these findings indicate that MAC-infected macrophages induced the expression of immune/inflammatory response genes, similar to the case in M1 activation, as expected from a previous study (28). In contrast, the expression profile of *Nos2* in MAC infection was quite different from those in IFNγ stimulation, Mtb infection, and MALP2 stimulation, suggesting that MAC infection induces M1-like, but distinct, macrophage activation.

### Expression profiles of M1- or M2-associated transcription factor genes in MAC-infected macrophages

**Figure 2:**
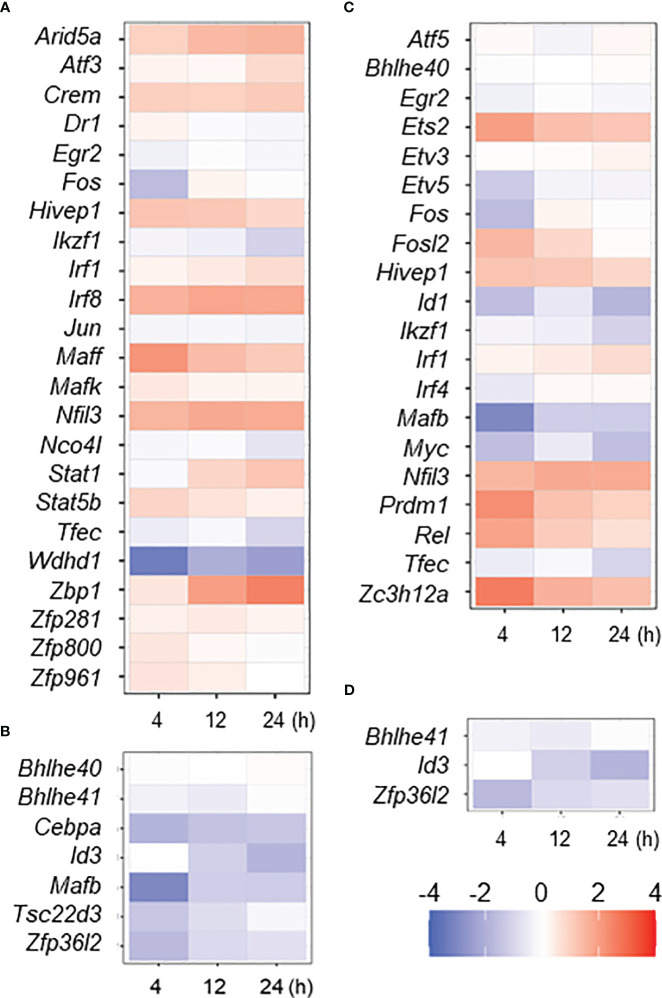
Expressional change of M1- or M2-associated transcription factor (TF) genes in MAC-infected macrophage. Up- or down-regulated TF genes in IFNγ- or IL4/IL13-stimulated macrophages (M1 or M2 activation, respectively) were taken from previous work (24), and their expressional change in MAC-infected macrophages was shown by heat map; IFNγ upregulated **(A)** and downregulated **(B)** and IL4/IL13 upregulated **(C)** and downregulated **(D)** TF genes. Degree of the change was shown by color of log2.

The gene expression dynamics in MAC infection underlays global changes of transcription factor (TF) gene expression. Because we previously described TF genes that are associated with IFNγ- or IL4/IL13-stimulated M1- or M2-activated macrophages, respectively (24), we examined how those TF genes are affected in MAC infection (**Figure 2**). We found that 17 out of 23 IFNγ-mediated M1-upregulated TF genes revealed significant upregulation in MAC infection (73.9%, **Figure 2A**), while *Egr2*, *Ikzf1*, *Jun*, *Noc4I*, *Tfec* and *Wdhd1* did not show the upregulation. For IFNγ-mediated M1-downregulated TF genes, 6 out of 7 TF genes revealed significant downregulation (85.7%, **Figure 2B**). The results indicate that majority of M1-associated TF genes was also associated with MAC infection.

Interestingly, we found that 7 out of 20 IL4/IL13-mediated M2-upregulated TF genes (*Ets2*, *Fosl2*, *Hivep1*, *Nfil3*, *Prdm1*, *Rel* and *Zc3h12a*) were significantly upregulated (35%) in MAC infection, while 7 TF genes (*Etv5*, *Fos*, *Id1*, *Ikzf1*, *Mafb*, *Myc* and *Tfec*) were not upregulated (**Figure 2C**). Two out of 3 IL4/IL13-mediated downregulated TF genes (*Id3* and *Zfp36I2*) were significantly downregulated in MAC infection (**Figure 2D**). Taken together, the results indicate again that MAC infection induces M1-like, but distinct, macrophage activation.

### LncRNAs in MAC-infected macrophages

**Figure 3:**
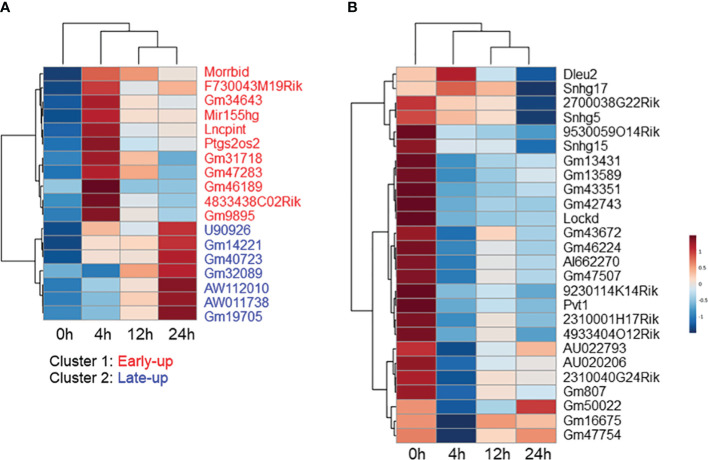
Expressional clustering analysis of long non-coding RNAs (lncRNAs) differentially expressed in *Mycobacterium avium* complex-infected macrophages. Hierarchical clustering of the expression profiles of **(A)** upregulated and **(B)** downregulated lncRNAs. Upregulated lncRNAs peaking at the early (4 h) stage were termed cluster 1 (red), and those peaking late (24 h) were termed cluster 2 (blue).

Non-coding RNAs play important roles in regulating gene expression in various ways. However, the function of the majority of them—in particular that of lncRNAs—is unknown. We explored differentially expressed lncRNA promoters in MAC-infected macrophages at each time point, and we identified 31 upregulated lncRNA promoters corresponding to 18 lncRNAs (**Table 1**) and 30 downregulated lncRNA promoters corresponding to 26 lncRNAs (**Table 2**). Expressional clustering analysis revealed that the upregulated lncRNAs were clustered into two groups that were upregulated early (4 h, cluster 1) or late (24 h, cluster 2) (**Figure 3A**). In contrast, the downregulated lncRNAs did not show distinct clusters (**Figure 3B**).

**Table 1:** LncRNAs upregulated in MAC-infected macrophages.

Cluster Name	Gene Symbol	Expression at 0h (cpm)	Fold change		
			4h/0h	12h/0h	24h/0h
p2@4833438C02Rik	4833438C02Rik	2.43	7.63	3.22	2.11
p1@AW011738	AW011738	1.46	1.66	2.88	4.53
p1@AW112010	AW112010	18.64	5.81	10.11	20.88
p2@AW112010	AW112010	4.77	2.63	5.31	9.72
p3@AW112010	AW112010	2.16	2.07	4.43	9.00
p4@AW112010	AW112010	0.94	1.87	3.51	12.33
p1@F730043M19Rik	F730043M19Rik	5.65	7.99	4.80	6.23
p2@F730043M19Rik	F730043M19Rik	2.16	6.28	4.60	5.52
p1@Gm14221	Gm14221	2.50	2.98	3.10	4.26
p1@Gm19705	Gm19705	0.37	3.77	9.11	18.85
p1@Gm31718	Gm31718	1.87	6.07	3.98	1.65
p1@Gm32089	Gm32089	22.90	0.74	1.80	2.23
p4@Gm32089	Gm32089	2.44	1.33	2.26	2.50
p1@Gm34643	Gm34643	2.27	5.50	3.47	3.15
p1@Gm40723	Gm40723	2.23	2.14	2.11	3.03
p1@Gm46189	Gm46189	2.78	3.45	0.97	0.96
p1@Gm47283	Gm47283	5.65	3.03	2.40	1.45
p5@Gm47283	Gm47283	1.46	3.33	2.55	1.40
p1@Gm9895	Gm9895	2.30	5.90	3.06	2.25
p1@Lncpint	Lncpint	12.69	2.57	1.71	1.79
p2@Lncpint	Lncpint	11.86	2.81	1.88	1.69
p3@Lncpint	Lncpint	16.45	2.75	1.45	0.85
p4@Lncpint	Lncpint	9.25	2.34	1.83	1.73
p9@Lncpint	Lncpint	1.48	4.14	1.77	1.36
p1@Mir155hg	Mir155hg	7.92	73.43	42.75	41.93
p2@Mir155hg	Mir155hg	0.25	36.01	18.91	14.31
p1@Morrbid	Morrbid	31.57	3.23	2.98	2.44
p3@Morrbid	Morrbid	17.16	2.13	1.91	1.59
p5@Morrbid	Morrbid	2.69	3.86	3.27	2.58
p1@Ptgs2os2	Ptgs2os2	1.55	5.95	2.74	3.06
p1@U90926	U90926	0.05	1121.89	800.36	1560.07

Statistically significant fold change values are shown in red font. The expanded **Table 1** with fold change values between other time points is shown in **Supplementary Table S11**.

**Table 2:** LncRNAs downregulated in MAC-infected macrophages.

Cluster Name	Gene Symbol	Expression at 0h (cpm)	Fold change		
			4h/0h	12h/0h	24h/0h
p2@2310001H17Rik	2310001H17Rik	15.70	0.27	0.67	0.59
p1@2310040G24Rik	2310040G24Rik	3.02	0.11	0.60	0.57
p1@2700038G22Rik	2700038G22Rik	4.91	0.74	0.70	0.23
p1@4933404O12Rik	4933404O12Rik	7.43	0.27	0.52	0.25
p1@9230114K14Rik	9230114K14Rik	5.42	0.31	0.43	0.27
p1@9530059O14Rik	9530059O14Rik	24.10	0.45	0.40	0.28
p1@AI662270	AI662270	19.05	0.24	0.54	0.45
p1@AU020206	AU020206	117.72	0.26	0.57	0.60
p2@AU020206	AU020206	85.09	0.24	0.57	0.61
p1@AU022793	AU022793	81.52	0.32	0.66	0.81
p2@AU022793	AU022793	18.48	0.18	0.39	0.50
p3@AU022793	AU022793	10.06	0.12	0.34	0.58
p2@Dleu2	Dleu2	10.91	0.42	0.33	0.25
p1@Gm13431	Gm13431	11.71	0.15	0.32	0.37
p1@Gm13589	Gm13589	7.53	0.16	0.31	0.43
p1@Gm16675	Gm16675	5.89	0.27	0.98	0.91
p1@Gm42743	Gm42743	965.78	0.14	0.25	0.22
p1@Gm43351	Gm43351	2.99	0.19	0.27	0.35
p1@Gm43672	Gm43672	6.70	0.29	0.68	0.50
p1@Gm46224	Gm46224	53.64	0.35	0.59	0.49
p1@Gm47507	Gm47507	41.72	0.15	0.50	0.39
p1@Gm47754	Gm47754	2.67	0.25	0.83	0.98
p1@Gm50022	Gm50022	5.59	0.32	0.64	1.15
p2@Gm50022	Gm50022	6.89	0.23	0.22	0.40
p1@Gm807	Gm807	6.01	0.16	0.59	0.50
p1@Lockd	Lockd	9.53	0.03	0.10	0.14
p1@Pvt1	Pvt1	12.28	0.09	0.27	0.13
p1@Snhg15	Snhg15	28.64	0.51	0.51	0.18
p2@Snhg17	Snhg17	7.96	0.65	0.78	0.27
p1@Snhg5	Snhg5	21.34	0.84	0.78	0.36

Statistically significant fold change values are shown in red font. The expanded **Table 2** with fold change values between other time points is shown in **Supplementary Table S12**.

**Figure 4:**
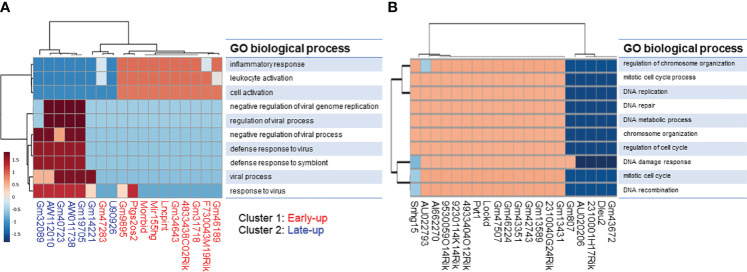
Gene ontology (GO) enrichment analysis of protein-coding transcripts associated with long non-coding RNAs. We extracted protein-coding transcripts with Pearson correlation coefficients of both more than 0.8 and less than -0.8 with each differentially expressed lncRNA, and we then subjected them to GO analysis. The top 10 most enriched GO biological processes of **(A)** upregulated and **(B)** downregulated lncRNA-associated protein-coding transcripts generated by using the ToppCluster tool (https://toppcluster.cchmc.org/).

We tried to interpret the biological role of the differentially expressed lncRNAs by performing a knowledge-based pathway analysis of the protein-coding transcripts that exhibited expressional correlation with each lncRNA. This was based on the assumption that the expression profiles of transcripts with similar roles are highly correlated with each other owing to their similar regulation, while those with opposite roles are anti-correlated owing to their opposite regulation. We extracted protein-coding transcripts with Pearson correlation coefficients of both more than 0.8 and less than -0.8 with each differentially expressed lncRNA, and we then subjected them to GO analysis. A summary of the GO biological process term similarity among the 18 upregulated lncRNAs (**Supplementary Figure S3A**) and the top 20 downregulated lncRNAs (**Supplementary Figure S3B**) was revealed by heatmap. This analysis showed that clusters 1 and 2 of the upregulated lncRNAs were associated with distinct GO terms. In contrast, the majority of downregulated lncRNAs were associated with similar GO terms. Analysis of the top 10 GO biological process terms showed that most of the upregulated lncRNAs in cluster 1 were associated with terms associated with immune activation, such as “inflammatory response,” “leukocyte activation,” and “cell activation,” whereas the upregulated lncRNAs in cluster 2 were associated with terms related to the immune response to infection, such as “negative regulation of viral genome replication,” “regulation of viral process,” “negative regulation of viral process,” and “defense response to virus” (**Figure 4A**). In contrast, the majority of the downregulated lncRNAs were weakly associated with pro-proliferation terms, such as “regulation of chromosome organization,” “mitotic cell-cycle process,” “DNA replication,” “DNA repair,” and “DNA damage response” (**Figure 4B**).

**Figure 5:**
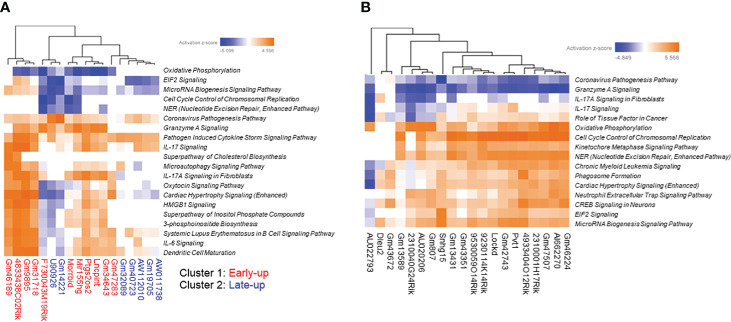
Prediction of canonical pathways associated with differentially expressed long non-coding RNAs (lncRNAs). We extracted protein-coding transcripts with Pearson correlation coefficients of both more than 0.9 and less than -0.9 with each differentially expressed lncRNA, and we then subjected them to Ingenuity Pathway Analysis (IPA) to predict canonical pathways associated with differentially expressed lncRNAs. The top most enriched canonical pathways of **(A)** upregulated and **(B)** downregulated lncRNA-associated protein-coding transcripts with z-scores greater than 4 generated by using the IPA platform.

The correlation dataset was also subjected to IPA analysis to predict the canonical pathways and upstream transcription regulators associated with differentially expressed lncRNAs. We found that the upregulated lncRNAs were positively associated with the canonical pathways “IL-17 Signaling” and “Pathogen Induced Cytokine Storm Signaling pathway” (**Figure 5A**). Furthermore, the pathways “Granzyme A Signaling” and “IL-17A Signaling in Fibroblasts” were strongly positively associated with cluster 1 upregulated lncRNAs but not with cluster 2 upregulated lncRNAs. Interestingly, the latter pathways were negatively associated with downregulated lncRNAs (**Figure 5B**). As expected from the GO analysis, downregulated lncRNAs were positively associated with the pathways “Cell Cycle Control of Chromosomal Replication,” “Kinetochore Metaphase Signaling Pathway,” and “NER (Nucleotide Excision Repair, Enhanced Pathway).”

**Figure 6:**
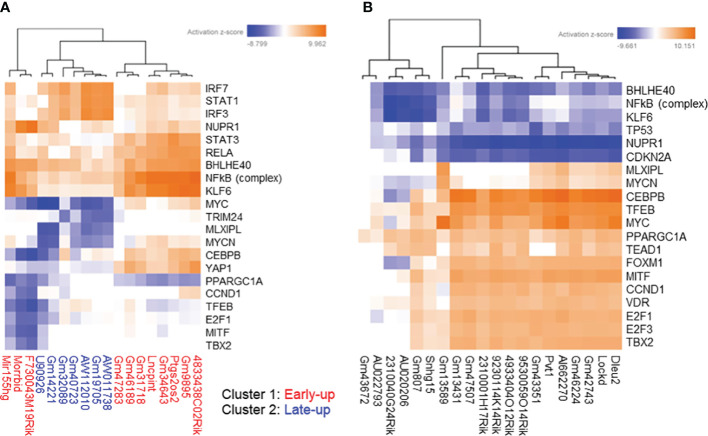
Prediction of upstream transcription regulators associated with differentially expressed long non-coding RNAs (lncRNAs). We extracted protein-coding transcripts with Pearson correlation coefficients of both more than 0.9 and less than -0.9 with each differentially expressed lncRNA, and we then subjected them to Ingenuity Pathway Analysis (IPA) to predict upstream transcription regulators associated with differentially expressed lncRNAs. The top most enriched upstream transcription regulators of **(A)** upregulated and **(B)** downregulated lncRNA-associated protein-coding transcripts with z-scores greater than 5 generated by using the IPA platform.

In the upstream transcription regulator analysis, cluster 1 upregulated lncRNAs were highly positively associated with STAT3, RELA, BHLHE40, NFkB (complex), and KLF6, whereas cluster 2 upregulated lncRNAs were highly positively associated with IRF7, STAT1, and IRF3 (**Figure 6A**). The downregulated lncRNAs were positively associated with proliferation-related transcription factors such as MYC and CEBPB, and negatively associated with inflammation-related transcription factors such as NFkB (complex) as upstream transcription regulators (**Figure 6B**).

Taken together, the results of our prediction analysis suggest that the differentially expressed lncRNAs are involved in the responses of MAC-infected macrophages, such as immune activation, immune response to infection, and proliferation.

### Literature analysis of lncRNAs differentially expressed in MAC-infected macrophages

**Figure 7:**
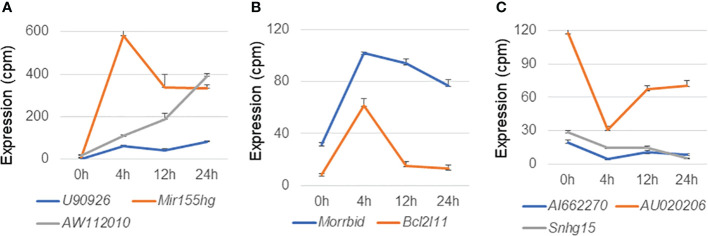
Expression profiles of representative long non-coding RNAs in macrophages infected with *Mycobacterium avium* complex. CAGE-seq transcriptome analysis was performed at 0, 4, 12, or 24 h post-infection. Average expression values and their standard deviation were shown in each figure. **(A)** Induction of representative lncRNAs, *U90926*, *Mir155hg*, and *AW112010*. **(B)** Expressional change of another upregulated lncRNA *Morrbid* and its associated protein-coding gene *Bcl2l11*. **(C)** Suppression of representative lncRNAs, *AI662270*, *AU020206*, and *Snhg15*.

Next, we explored literature citations of the differentially expressed lncRNAs individually. Seven upregulated lncRNAs had at least one citation, and among them *U90926*, *Mir155hg*, *AW112010*, and *Morrbid* were of particular interest (**Figures 7A, B**). It has recently been reported that expression of the lncRNA *U90926* is induced in activated macrophages and is protective in endotoxic shock, and that this lncRNA encodes a novel secreted protein (29). In the case of *Mir155hg*, a MiR-155-regulated molecular network orchestrates cell fate in the innate and adaptive immune response to Mtb (30). Furthermore, translation of the non-canonical open reading frames of *AW112010* controls mucosal immunity (31), and the lncRNA *Morrbid* negatively regulates the transcription of protein-coding gene *Bcl2l11* and the lifespans of short-lived myeloid cells (32). In fact, we observed a radical decline in *Bcl2l11* expression when *Morrbid* was upregulated (**Figure 7B**). Among the downregulated lncRNAs, eight had at least one citation; *AI662270*, *AU020206*, and *Snhg15* were of particular interest (**Figure 7C**), as it has been reported that *AI662270* and *AU020206* are involved in the atherosclerosis induced by macrophages (33, 34) and *Snhg15* is involved in inflammation (35).

### Effect of M1/M2 preactivation on MAC-infection-mediated differentially expressed lncRNAs

**Figure 8:**
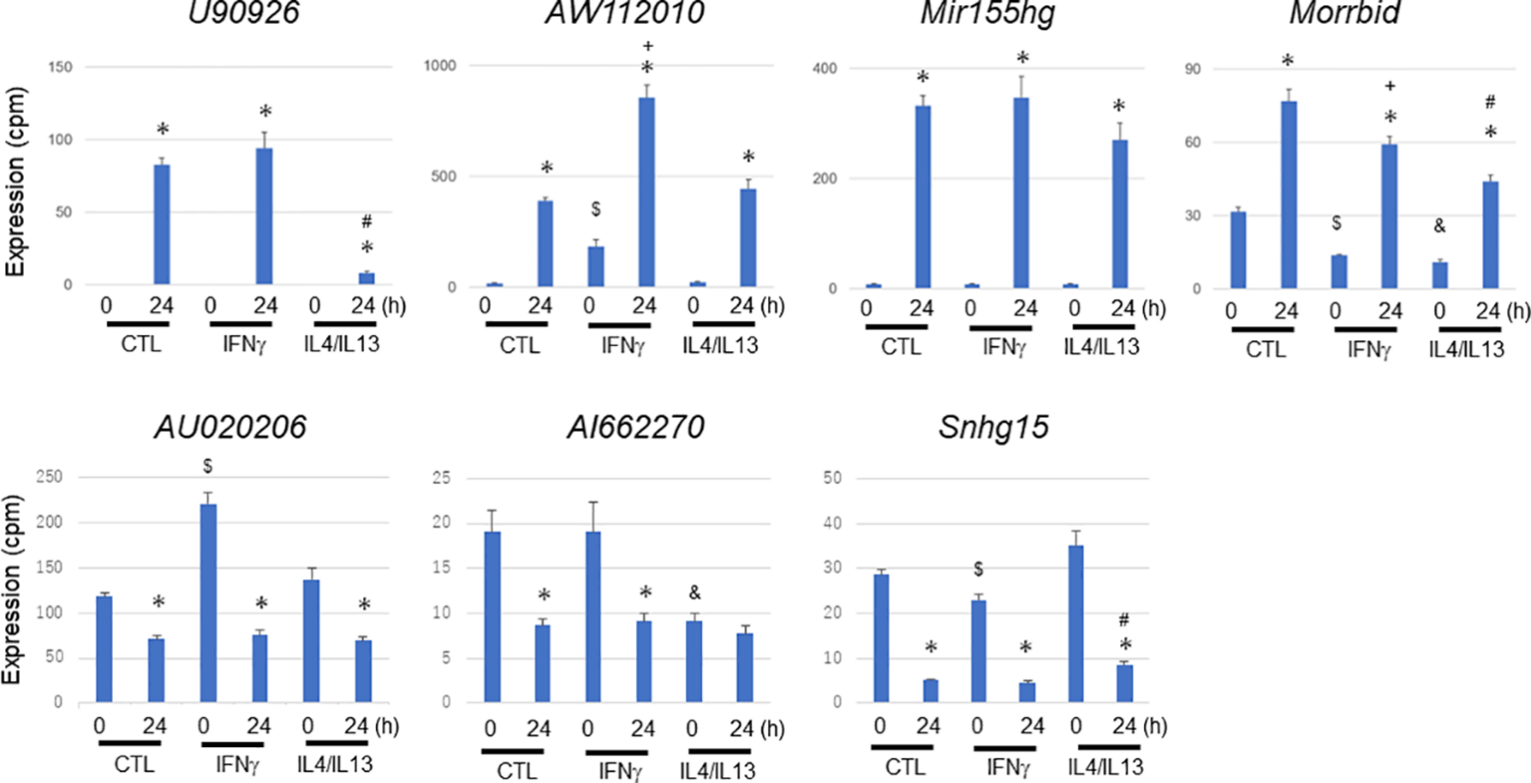
Expressional change of representative long non-coding RNAs during *Mycobacterium avium* complex infection under no preactivation (CTL) or under M1 (IFNγ) or M2 (IL4/IL13) preactivation. IFNγ or IL4/IL13 stimulation was carried out 24 h prior to *Mycobacterium avium* complex infection (M1 or M2 preactivation). After 0 and 24 h of the infection, total RNA was extracted from macrophages and subjected to CAGE-seq transcriptome analysis. Average expression values and their standard deviation were shown in each figure. *, #, $, +, and & denote statistical significance between 0 and 24 h, between no- and M2-preactivation at 24 h, between no- and M1-preactivation at 0 h, between no- and M1-preactivation at 24 h, and between no- and M2-preactivation at 0 h, respectively (P value less than 0.01 with Student’s unpaired T test).

To characterize the abovementioned differentially expressed lncRNAs of interest, we further explored their expression profiles in MAC-infected macrophages under IFNγ-mediated M1 preactivation and Il4/IL13-mediated M2 preactivation (**Figure 8**). MAC-infection-mediated upregulation of *U90926* was dramatically suppressed by M2 preactivation. In contrast, *AW112010* upregulation was enhanced by M1 preactivation, which is similar to the expression profile of *Nos2* (**Supplementary Figure S4**) suggesting that *AW112010*, as well as *Nos2,* is synergistically regulated by IFNγ-Jak/Stat and Tlr2-Nfkb pathways. On the other hand, *Mir155hg* upregulation seemed to be unaffected by M1 or M2 preactivation and *Morrbid* showed complicated expressional change. In the case of the downregulated lncRNAs, *AU020206* was upregulated by M1 preactivation, but this enhancement was downregulated upon MAC infection. Furthermore, *AI662270* expression was suppressed by M2 preactivation, whereas *Snhg15* was affected by M1 or M2 preactivation in a complicated manner. These results indicated that the lncRNAs underwent a variety of expressional changes upon M1 or M2 preactivation and subsequent MAC infection, implying the distinct role of each lncRNA in MAC infection under macrophage polarization.

### Signal transduction of MAC-infected macrophages for lncRNA regulation

**Figure 9:**
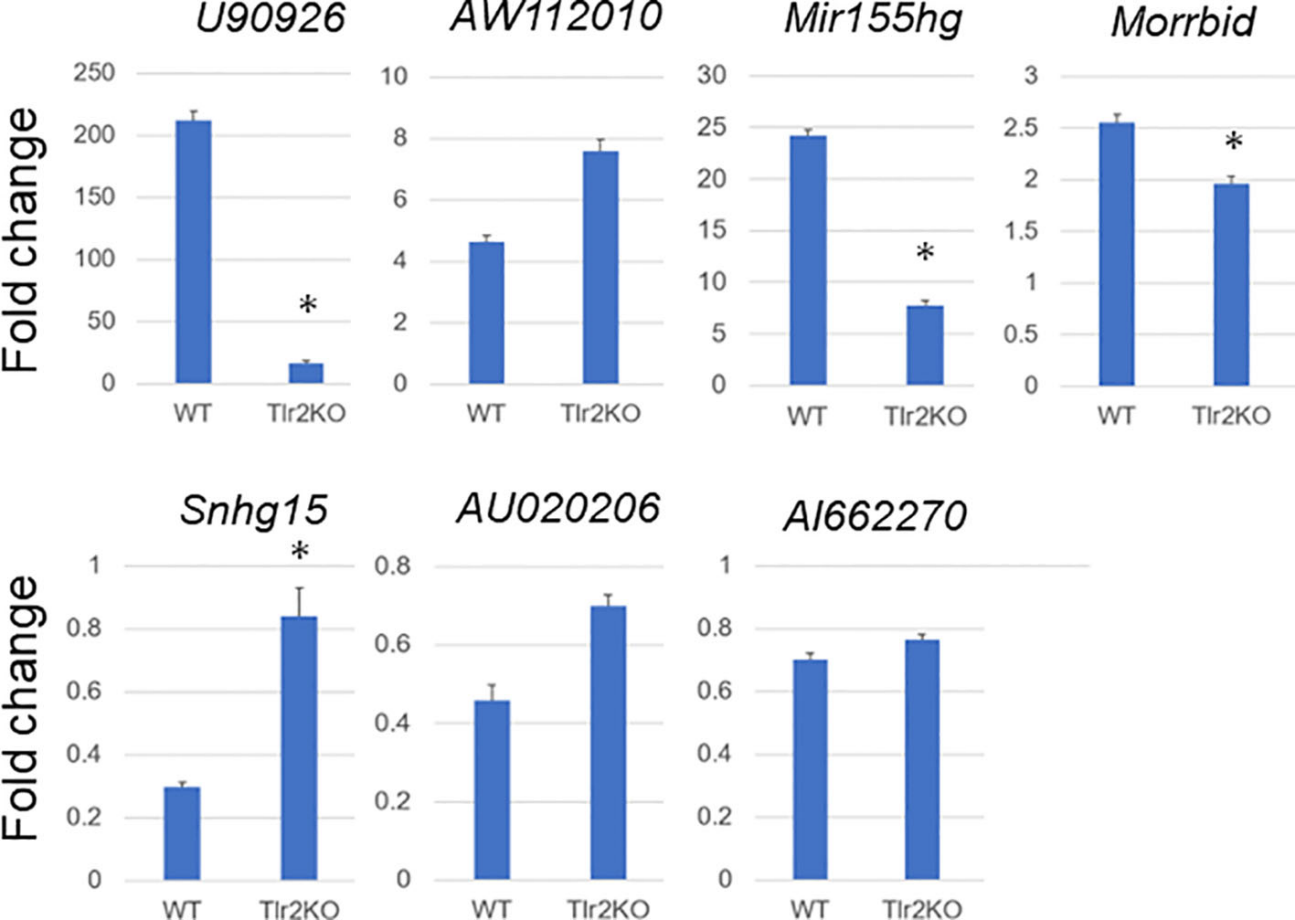
Expressional fold change of representative long non-coding RNAs in *Mycobacterium avium* complex-infected wild-type (WT) and Tlr2 knockout (Tlr2KO) macrophages. Expressional fold change of representative long non-coding RNAs between 0 and 24 h of *Mycobacterium avium* complex-infected WT and Tlr2KO macrophages was analyzed by RT-qPCR. Average expressional fold change and its standard deviation were shown in each figure. Asterisks denote P value less than 0.01 with Student’s unpaired T test between WT and Tlr2KO macrophages.

Tlr2 and Tlr4 play essential roles in signal transduction in bacterial infection. Tlr2/4 promotes the expression of inflammatory cytokine and chemokine genes via a myeloid differentiation primary response 88 (Myd88)-mediated signal transduction pathway, and Tlr4 promotes the expression of other inflammatory genes via an Myd88-independent signal transduction pathway (36). MAC is predominantly sensed by Tlr2 (27), although the existence of other sensing mechanisms has been suggested (37). To evaluate whether the expressional change of lncRNAs in MAC-infected macrophages is mediated by the Tlr2 signal transduction pathway, we performed an MAC infection experiment by using BMDMs derived from Tlr2 knockout mice. After confirming the absence of *Tlr2* expression in BMDMs derived from Tlr2 knockout mice (**Supplementary Figure S5**), we subjected total RNA extracted from MAC-infected wild-type and Tlr2 knockout BMDMs to RT-qPCR analysis (**Figure 9**). Upregulation of the lncRNAs *U90926*, *Mir155hg*, and *Morrbid* was suppressed in BMDMs derived from *Tlr2* knockout mice. *AW112010* expression was not suppressed. The MAC-infection-mediated downregulation of *Snhg15* seen in wild-type BMDMs was suppressed in BMDMs derived from *Tlr2* knockout mice. On the other hand, most of dramatic upregulation of cytokine and chemokine genes, and *Nos2* and *Arg1*, was suppressed in BMDMs derived from Tlr2 knockout mice (**Supplementary Figure S6**). Finally, we used MALP2 stimulation to confirm the Tlr2-mediated upregulation or suppression of these lncRNAs (**Supplementary Figure S7**). Taken together, the results indicate that the expressional change of many lncRNAs in MAC-infected BMDMs is mediated by Tlr2, although there may be other mechanisms for sensing MAC infection.

## Discussion

We performed a comprehensive transcriptome analysis of MAC-infected macrophages *in vitro*. In addition to analyzing the expression of protein-coding immune/inflammatory response genes and *Nos2*/*Arg1* genes, we successfully identified lncRNAs that were differentially expressed in a time-dependent manner after MAC infection. We also predicted the functions, canonical pathways, and upstream transcription regulators of the differentially expressed lncRNAs from those of protein-coding genes with similar expression profiles. Furthermore, we showed that representative lncRNAs underwent various expressional changes upon M1 or M2 preactivation and subsequent MAC infection. Finally, by using Tlr2 knockout BMDMs, we showed that the expressional change of part of those lncRNAs was mediated by Tlr2. The availability of high-resolution transcriptomic data on MAC-infected macrophages has been sparse. In particular, to our knowledge, the dynamics of lncRNAs in MAC-infected macrophages have not been studied before. Therefore, the data obtained here are very valuable for understanding the host response to MAC infection.

MAC-infected BMDMs underwent the dramatic induction of inflammation genes such as cytokines and chemokines 4 h after infection, although *Arg1*, an M2 marker gene, continued to be upregulated 24 h after infection. This could have represented a rapid transition of macrophage activation from M1 to M2 during MAC infection. However, the inflammation genes characteristic of M1 activation also continued to be highly expressed 24 h after MAC infection. Furthermore, we did not detect upregulation of the M2 marker genes *Lgals3* and *Mrc1* in the 24 h after MAC infection. Therefore, we can say that MAC-infected BMDMs show M1-like activation for at least 24 h after infection. Importantly, transient induction of *Nos2*, an M1 marker gene, was not observed in MAC-infected macrophages, although *Nos2* expression was induced transiently and dominantly upon IFNγ-mediated M1 activation and Mtb infection. Furthermore, transient *Nos2* induction was observed in response to the Tlr2 agonist MALP2, although MAC is known to be sensed predominantly by Tlr2 (27). The results suggest that, besides the major Tlr2-mediated sensing pathway, there is another, unidentified, pathway or pathways by which MAC is sensed and that strongly inhibits transient *Nos2* induction.

We found that an MOI of 200 was required for MAC infection to produce a sufficient immune/inflammation response to macrophages. This is a relatively high value compared with the MOI of only 5 for Mtb infection (17). The specific *Nos2/Arg1* profiles in MAC-infected BMDMs, together with the weak sensing properties of BMDMs for MAC infection, must be advantageous for MAC to survive in macrophages, as Nos2-mediated nitric oxide production—one of the strong microbicidal activities of macrophages—is low in MAC infection. A future exploration of the molecular mechanism by which *Nos2* transient induction is suppressed in MAC-infected BMDMs may provide hints for the treatment of MAC infection.

lncRNAs regulate gene expression in various ways. The differentially expressed lncRNAs identified in MAC-infected macrophages may be involved in regulating the expression of various target genes in response to MAC infection. Our finding that each lncRNA is uniquely regulated by M1 or M2 preactivation and subsequent MAC infection suggests the diversity of the functions of these lncRNAs. We found that upregulated lncRNAs could be categorized into two clusters, with early-upregulated lncRNAs being associated with immune activation and late-upregulated lncRNAs being associated with the immune response to infection. The predicted canonical pathways and upstream transcription regulators, derived from the IPA analysis, also supported inference of the GO analysis. However, all of our predictions were based on the analysis of protein-coding genes with similar expression kinetics. Further studies are needed to elucidate the detailed functions of each differentially expressed lncRNA.

To analyze signal transduction pathways for the expressional change of lncRNAs in MAC infection, we explored changes in their expression by using Tlr2 knockout BMDMs. The MAC infection-mediated upregulation of the lncRNAs *U90926*, *Mir155hg*, and *Morrbid* and downregulation of the lncRNAs *Snhg15* revealed the apparent suppression of expressional change (**Figure 9**), and this is consistent with previous reports that MAC is sensed predominantly by Tlr2 (27). However, lncRNAs with relatively mild expressional change in MAC infection (*AW112010*, *AU020206*, and *AI662270*) did not show obvious suppression of up- or downregulation in Tlr2 knockout BMDMs, although the responses to MALP2 (**Supplementary Figure S7**) indicated that at least part of the expressional change of these genes was mediated by Tlr2. This suggests that there is another mechanism for sensing MAC infection. In fact, Tlr9 is another known sensing mechanism that recognizes the non-methylated CpG DNA of *M. avium* (38). Furthermore, Tlr4 and Tlr6 may be involved in other *M. avium-*sensing mechanisms (37, 39). Whether the expressional change of *AW112010*, *AU020206*, and *AI662270* is regulated by another Tlr-mediated MAC sensing mechanism is an interesting issue for future exploration.

Here, we performed a comprehensive transcriptome analysis of MAC-infected macrophages and successfully identified lncRNAs that were affected by the infection. Because the experimental system used in this study was an *in vitro* model in which macrophage cells were infected with high MOI of MAC, the interpretation of the results should be done with caution. Now that our knowledge of the transcriptome in mammals has been increasing, information on lncRNAs will be essential to our overall understanding of host immune responses to NTMs. We have merely paved the way: Further analysis is necessary to elucidate the whole picture and thus enable the development of treatments for MAC syndrome.

## Data availability statement

The datasets presented in this study can be found in online repositories. The names of the repository/repositories and accession number(s) can be found below: https://www.ncbi.nlm.nih.gov/, GSE 249460.

## Ethics statement

The animal study was approved by The Animal Care and Use Committee of the National Institute of Infectious Diseases. The study was conducted in accordance with the local legislation and institutional requirements.

## Author contributions

MY: Investigation, Methodology, Writing – original draft, Writing – review & editing. ATK: Data curation, Formal analysis, Writing – review & editing. XQ: Data curation, Formal analysis, Visualization, Writing – review & editing. HN: Formal analysis, Investigation, Writing – review & editing. SM: Investigation, Visualization, Writing – review & editing. YM: Investigation, Resources, Writing – review & editing. YY: Resources, Writing – review & editing. YH: Conceptualization, Funding acquisition, Project administration, Supervision, Writing – original draft, Writing – review & editing. HS: Conceptualization, Project administration, Supervision, Writing – original draft, Writing – review & editing.

## Supplementary material

Supplementary Figure 1 Determination of multiplicity of infection (MOI) in an *Mycobacterium avium* complex (MAC) infection experiment. Bone-marrow-derived macrophages were infected with MAC at MOIs of 0, 5, 50, and 500. After 4 h of incubation, total RNA was extracted from harvested cells. This was followed by RT-qPCR measurement of the *Il1a*, *Il10*, and *Cxcl2* genes. The fold change of expression of the genes at each MOI was calculated in comparison with an MOI of 0. Average results of duplicate experiments are shown.

Supplementary Figure 2 Expressional change of M1-activated genes upon IFNγ stimulation, *Mycobacterium tuberculosis* (Mtb) infection, and MALP2 stimulation. Gene expression profiles for **(A)** cytokines, **(B)** chemokines, **(C)** *Nos2*, and **(D)** *Arg1* in IFNγ-stimulated bone-marrow derived macrophages (BMDMs) and **(E)** of *Nos2* and **(F)** *Arg1* in Mtb-infected BMDMs. Average values of triplicate data are shown. The standard deviation is also shown in figures **(C–F)**. The expression profiles were taken from the FANTOM5 database (40). The fold change of expression of *Nos2* and *Arg1* stimulated by the Tlr2 ligand MALP2 was calculated in comparison with no stimulation (**(G, H)**, respectively) by RT-qPCR. Average results of duplicate experiments are shown. Expression profiles of two M2 marker genes, *Lgals3* and *Mrc1*, in *Mycobacterium avium* complex-infected macrophages are also shown **(I)**. Average expression values of quadruplicate data were plotted, but the standard deviation omitted.

Supplementary Figure 3 Clustering analysis of predicted gene ontology (GO) biological process terms for long non-coding RNA (lncRNA)-associated protein-coding transcripts. We extracted protein-coding transcripts with Pearson correlation coefficients of both more than 0.8 and less than -0.8 with each differentially expressed lncRNA, and we then subjected them to GO analysis. Hierarchical clustering of the Pearson correlation coefficients of log-transformed *P*-values for GO biological process terms associated with **(A)** upregulated and **(B)** downregulated lncRNA-associated protein-coding transcripts.

Supplementary Figure 4 Expressional change of *Nos2* during *Mycobacterium avium* complex infection under no preactivation (CTL) or under M1 (IFNγ) preactivation. IFNγ stimulation was carried out 24 h prior to *Mycobacterium avium* complex (MAC) infection (M1 preactivation). After 0 and 24 h of the infection, total RNA was extracted from macrophages and subjected to CAGE-seq transcriptome analysis. Asterisks denote P value less than 0.01 with Student’s unpaired T test.

Supplementary Figure 5 Expression of Tlr2 genes. *Tlr2* expression in wild-type (WT) and Tlr2 knockout (Tlr2KO) mouse bone-marrow derived macrophages (BMDMs) was measured by RT-qPCR to confirm that Tlr2KO BMDMs did not express *Tlr2*.

Supplementary Figure 6 Expressional fold change of several protein-coding genes in *Mycobacterium avium* complex-infected wild-type (WT) and Tlr2 knockout (Tlr2KO) macrophages by RT-qPCR. Expressional fold change for indicated genes was calculated between 0 and 24 h after *Mycobacterium avium* complex infection in WT and Tlr2KO macrophages by RT-qPCR. Asterisks denote P value less than 0.01 with Student’s unpaired T test between WT and Tlr2KO macrophages.

Supplementary Figure 7 Expressional fold change of representative long non-coding RNAs upon MALP2 stimulation. Expressional fold change for seven representative lncRNAs was calculated between 0 and 4 h after MALP2 stimulated macrophages by RT-qPCR. Average results of duplicate experiments are shown.

Supplementary Table 1 Promoter-level gene expression table of *Mycobacterium avium* complex-infected macrophages. Expression levels are shown as tags per million (CPM).

Supplementary Table 2 Genes up- and downregulated in *Mycobacterium avium* complex-infected macrophages at 4 h.

Supplementary Table 3 Genes up- and downregulated in *Mycobacterium avium* complex-infected macrophages at 12 h.

Supplementary Table 4 Genes up- and downregulated in *Mycobacterium avium* complex-infected macrophages at 24 h.

Supplementary Table 5 Gene ontology analysis of genes upregulated in *Mycobacterium avium* complex-infected macrophages at 4 h.

Supplementary Table 6 Gene ontology analysis of genes upregulated in *Mycobacterium avium* complex-infected macrophages at 12 h.

Supplementary Table 7 Gene ontology analysis of genes upregulated in *Mycobacterium avium* complex-infected macrophages at 24 h.

Supplementary Table 8 Gene ontology analysis of genes downregulated in *Mycobacterium avium* complex-infected macrophages at 4 h.

Supplementary Table 9 Gene ontology analysis of genes downregulated in *Mycobacterium avium* complex-infected macrophages at 12 h.

Supplementary Table 10 Gene ontology analysis of genes downregulated in *Mycobacterium avium* complex-infected macrophages at 24 h.

Supplementary Table 11 Expanded with fold change values between other time points.

Supplementary Table 12 Expanded with fold change values between other time points.

## References

[1] Namkoong H Kurashima A Morimoto K Hoshino Y Hasegawa N Ato M. Epidemiology of pulmonary nontuberculous mycobacterial disease, Japan. Emerg Infect Dis. (2016) 22:1116–7. doi: 10.3201/eid2206.151086 PMC4880076 27191735

[2] Prevots DR Marshall JE Wagner D Morimoto K. Global epidemiology of nontuberculous mycobacterial pulmonary disease: A review. Clin Chest Med. (2023) 44:675–721. doi: 10.1016/j.ccm.2023.08.012 37890910 PMC10625169

[3] Shah NM Davidson JA Anderson LF Lalor MK Kim J Thomas HL. Pulmonary *Mycobacterium avium*-intracellulare is the main driver of the rise in non-tuberculous mycobacteria incidence in England, Wales and Northern Ireland, 2007-2012. BMC Infect Dis. (2016) 16:195. doi: 10.1186/s12879-016-1521-3 27154015 PMC4858927

[4] Thomson RM Centre NTMwgaQTC Queensland Mycobacterial Reference L. Changing epidemiology of pulmonary nontuberculous mycobacteria infections. Emerg Infect Dis. (2010) 16:1576–83. doi: 10.3201/eid1610.091201 PMC3294381 20875283

[5] Winthrop KL Marras TK Adjemian J Zhang H Wang P Zhang Q. Incidence and prevalence of nontuberculous mycobacterial lung disease in a large U.S. Managed care health plan, 2008-2015. Ann Am Thorac Soc. (2020) 17:178–85. doi: 10.1513/AnnalsATS.201804-236OC PMC6993793 31830805

[6] Hoefsloot W van Ingen J Andrejak C Angeby K Bauriaud R Bemer P. The geographic diversity of nontuberculous mycobacteria isolated from pulmonary samples: an NTM-NET collaborative study. Eur Respir J. (2013) 42:1604–13. doi: 10.1183/09031936.00149212 23598956

[7] Morimoto K Hasegawa N Izumi K Namkoong H Uchimura K Yoshiyama T. A laboratory-based analysis of nontuberculous mycobacterial lung disease in Japan from 2012 to 2013. Ann Am Thorac Soc. (2017) 14:49–56. doi: 10.1513/AnnalsATS.201607-573OC 27788025

[8] Kumar K Loebinger MR. Nontuberculous mycobacterial pulmonary disease: clinical epidemiologic features, risk factors, and diagnosis: the nontuberculous mycobacterial series. Chest. (2022) 161:637–46. doi: 10.1016/j.chest.2021.10.003 34627854

[9] Jenner RG Young RA. Insights into host responses against pathogens from transcriptional profiling. Nat Rev Microbiol. (2005) 3:281–94. doi: 10.1038/nrmicro1126 15806094

[10] Agdestein A Jones A Flatberg A Johansen TB Heffernan IA Djonne B. Intracellular growth of *Mycobacterium avium* subspecies and global transcriptional responses in human macrophages after infection. BMC Genomics. (2014) 15:58. doi: 10.1186/1471-2164-15-58 24450835 PMC3906092

[11] Matsuyama M Martins AJ Shallom S Kamenyeva O Kashyap A Sampaio EP. Transcriptional response of respiratory epithelium to nontuberculous mycobacteria. Am J Respir Cell Mol Biol. (2018) 58:241–52. doi: 10.1165/rcmb.2017-0218OC PMC5806000 28915071

[12] Nakajima M Matsuyama M Kawaguchi M Kiwamoto T Matsuno Y Morishima Y. Nrf2 Regulates Granuloma Formation and Macrophage Activation during *Mycobacterium avium* Infection via Mediating Nramp1 and HO-1 Expressions. mBio. (2021) 12:e01947-20. doi: 10.1128/mBio.01947-20 33563837 PMC7885113

[13] Mattick JS Amaral PP Carninci P Carpenter S Chang HY Chen LL. Long non-coding RNAs: definitions, functions, challenges and recommendations. Nat Rev Mol Cell Biol. (2023) 24:430–47. doi: 10.1038/s41580-022-00566-8 PMC10213152 36596869

[14] Schmerer N Schulte LN. Long noncoding RNAs in bacterial infection. Wiley Interdiscip Rev RNA. (2021) 12:e1664. doi: 10.1002/wrna.1664 33989449

[15] Uchiya K Takahashi H Yagi T Moriyama M Inagaki T Ichikawa K. Comparative genome analysis of *Mycobacterium avium* revealed genetic diversity in strains that cause pulmonary and disseminated disease. PloS One. (2013) 8:e71831. doi: 10.1371/journal.pone.0071831 23990995 PMC3749206

[16] Murata M Nishiyori-Sueki H Kojima-Ishiyama M Carninci P Hayashizaki Y Itoh M. Detecting expressed genes using CAGE. Methods Mol Biol. (2014) 1164:67–85. doi: 10.1007/978-1-4939-0805-9_7 24927836

[17] Roy S Schmeier S Kaczkowski B Arner E Alam T Ozturk M. Transcriptional landscape of *Mycobacterium tuberculosis* infection in macrophages. Sci Rep. (2018) 8:6758. doi: 10.1038/s41598-018-24509-6 29712924 PMC5928056

[18] Hasegawa A Daub C Carninci P Hayashizaki Y Lassmann T. MOIRAI: a compact workflow system for CAGE analysis. BMC Bioinf. (2014) 15:144. doi: 10.1186/1471-2105-15-144 PMC4033680 24884663

[19] Li H Durbin R. Fast and accurate short read alignment with Burrows-Wheeler transform. Bioinformatics. (2009) 25:1754–60. doi: 10.1093/bioinformatics/btp324 PMC2705234 19451168

[20] Robinson MD McCarthy DJ Smyth GK. edgeR: a Bioconductor package for differential expression analysis of digital gene expression data. Bioinformatics. (2010) 26:139–40. doi: 10.1093/bioinformatics/btp616 PMC2796818 19910308

[21] Kaimal V Bardes EE Tabar SC Jegga AG Aronow BJ. ToppCluster: a multiple gene list feature analyzer for comparative enrichment clustering and network-based dissection of biological systems. Nucleic Acids Res. (2010) 38:W96–102. doi: 10.1093/nar/gkq418 20484371 PMC2896202

[22] Kramer A Green J Pollard J Jr. Tugendreich S. Causal analysis approaches in Ingenuity Pathway Analysis. Bioinformatics. (2014) 30:523–30. doi: 10.1093/bioinformatics/btt703 PMC3928520 24336805

[23] Nishimura T Tamizu E Uno S Uwamino Y Fujiwara H Nishio K. hsa-miR-346 is a potential serum biomarker of *Mycobacterium avium* complex pulmonary disease activity. J Infect Chemother. (2017) 23:703–8. doi: 10.1016/j.jiac.2017.07.015 28827075

[24] Roy S Schmeier S Arner E Alam T Parihar SP Ozturk M. Redefining the transcriptional regulatory dynamics of classically and alternatively activated macrophages by deepCAGE transcriptomics. Nucleic Acids Res. (2015) 43:6969–82. doi: 10.1093/nar/gkv646 PMC4538831 26117544

[25] Misson P van den Brule S Barbarin V Lison D Huaux F. Markers of macrophage differentiation in experimental silicosis. J Leukoc Biol. (2004) 76:926–32. doi: 10.1189/jlb.0104019 15292275

[26] Sawada T Falk LA Rao P Murphy WJ Pluznik DH. IL-6 induction of protein-DNA complexes via a novel regulatory region of the inducible nitric oxide synthase gene promoter: role of octamer binding proteins. J Immunol. (1997) 158:5267–76. doi: 10.4049/jimmunol.158.11.5267 9164945

[27] Wang T Lafuse WP Zwilling BS. Regulation of toll-like receptor 2 expression by macrophages following *Mycobacterium avium* infection. J Immunol. (2000) 165:6308–13. doi: 10.4049/jimmunol.165.11.6308 11086067

[28] Redente EF Higgins DM Dwyer-Nield LD Orme IM Gonzalez-Juarrero M Malkinson AM. Differential polarization of alveolar macrophages and bone marrow-derived monocytes following chemically and pathogen-induced chronic lung inflammation. J Leukoc Biol. (2010) 88:159–68. doi: 10.1189/jlb.0609378 PMC2892523 20360403

[29] Sabikunnahar B Caldwell S Varnum S Hogan T Cooper A Lahue KG. Long noncoding RNA U90926 is induced in activated macrophages, is protective in endotoxic shock, and encodes a novel secreted protein. J Immunol. (2023) 210:807–19. doi: 10.4049/jimmunol.2200215 PMC9998366 36705532

[30] Rothchild AC Sissons JR Shafiani S Plaisier C Min D Mai D. MiR-155-regulated molecular network orchestrates cell fate in the innate and adaptive immune response to Mycobacterium tuberculosis. Proc Natl Acad Sci U S A. (2016) 113:E6172–E81. doi: 10.1073/pnas.1608255113 PMC5068277 27681624

[31] Jackson R Kroehling L Khitun A Bailis W Jarret A York AG. The translation of non-canonical open reading frames controls mucosal immunity. Nature. (2018) 564:434–8. doi: 10.1038/s41586-018-0794-7 PMC6939389 30542152

[32] Kotzin JJ Spencer SP McCright SJ Kumar DBU Collet MA Mowel WK. The long non-coding RNA Morrbid regulates Bim and short-lived myeloid cell lifespan. Nature. (2016) 537:239–43. doi: 10.1038/nature19346 PMC5161578 27525555

[33] Hong Y Zhang Y Chen H Tang X Zhao H Meng Z. Genetic dissection of the impact of lncRNA AI662270 during the development of atherosclerosis. J Transl Med. (2023) 21:97. doi: 10.1186/s12967-023-03962-6 36755320 PMC9906833

[34] Zhang C Zhang X Gong Y Li T Yang L Xu W. Role of the lncRNA-mRNA network in atherosclerosis using ox-low-density lipoprotein-induced macrophage-derived foam cells. Mol Omics. (2020) 16:543–53. doi: 10.1039/D0MO00077A 32915179

[35] Sun H Li S Xu Z Liu C Gong P Deng Q. SNHG15 is a negative regulator of inflammation by mediating TRAF2 ubiquitination in stroke-induced immunosuppression. J Neuroinflammation. (2022) 19:1. doi: 10.1186/s12974-021-02372-z 34980176 PMC8722265

[36] Kaisho T Takeuchi O Kawai T Hoshino K Akira S. Endotoxin-induced maturation of MyD88-deficient dendritic cells. J Immunol. (2001) 166:5688–94. doi: 10.4049/jimmunol.166.9.5688 11313410

[37] Marinho FA de Paula RR Mendes AC de Almeida LA Gomes MT Carvalho NB. Toll-like receptor 6 senses *Mycobacterium avium* and is required for efficient control of mycobacterial infection. Eur J Immunol. (2013) 43:2373–85. doi: 10.1002/eji.201243208 23716075

[38] Carvalho NB Oliveira FS Duraes FV de Almeida LA Florido M Prata LO. Toll-like receptor 9 is required for full host resistance to *Mycobacterium avium* infection but plays no role in induction of Th1 responses. Infect Immun. (2011) 79:1638–46. doi: 10.1128/IAI.01030-10 PMC3067546 21300776

[39] Lee KI Choi HG Son YJ Whang J Kim K Jeon HS. Mycobacterium avium MAV2052 protein induces apoptosis in murine macrophage cells through Toll-like receptor 4. Apoptosis. (2016) 21:459–72. doi: 10.1007/s10495-016-1220-y 26842846

[40] Forrest AR Kawaji H Rehli M Baille JK de Hoon MJ Haberle V. A promoter-level mammalian expression atlas. Nature. (2014) 507:462–70. doi: 10.1038/nature13182 PMC4529748 24670764

