# Supplementary figures and images for: Transcriptome analysis of long non-coding RNAs in Mycobacterium avium complex–infected macrophages
Source: Front Immunol. 2024 Apr 22;15:1374437. doi: 10.3389/fimmu.2024.1374437 (PMC11070510; doi:10.3389/fimmu.2024.1374437)

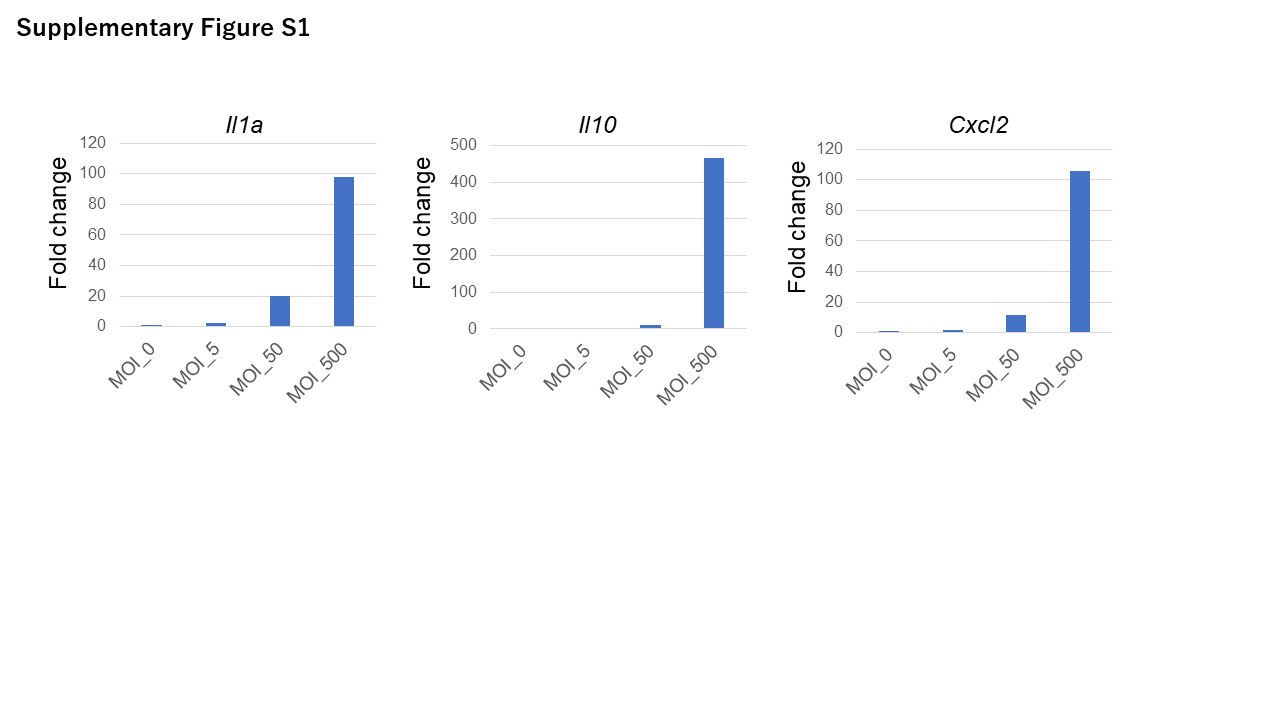

Supplement: Supplementary Figure 1 — Determination of multiplicity of infection (MOI) in an Mycobacterium avium complex (MAC) infection experiment. Bone-marrow-derived macrophages were infected with MAC at MOIs of 0, 5, 50, and 500. After 4 h of incubation, total RNA was extracted from harvested cells. This was followed by RT-qPCR measurement of the Il1a, Il10, and Cxcl2 genes. The fold change of expression of the genes at each MOI was calculated in comparison with an MOI of 0. Average results of duplicate experiments are shown. [file Image_1.tif]

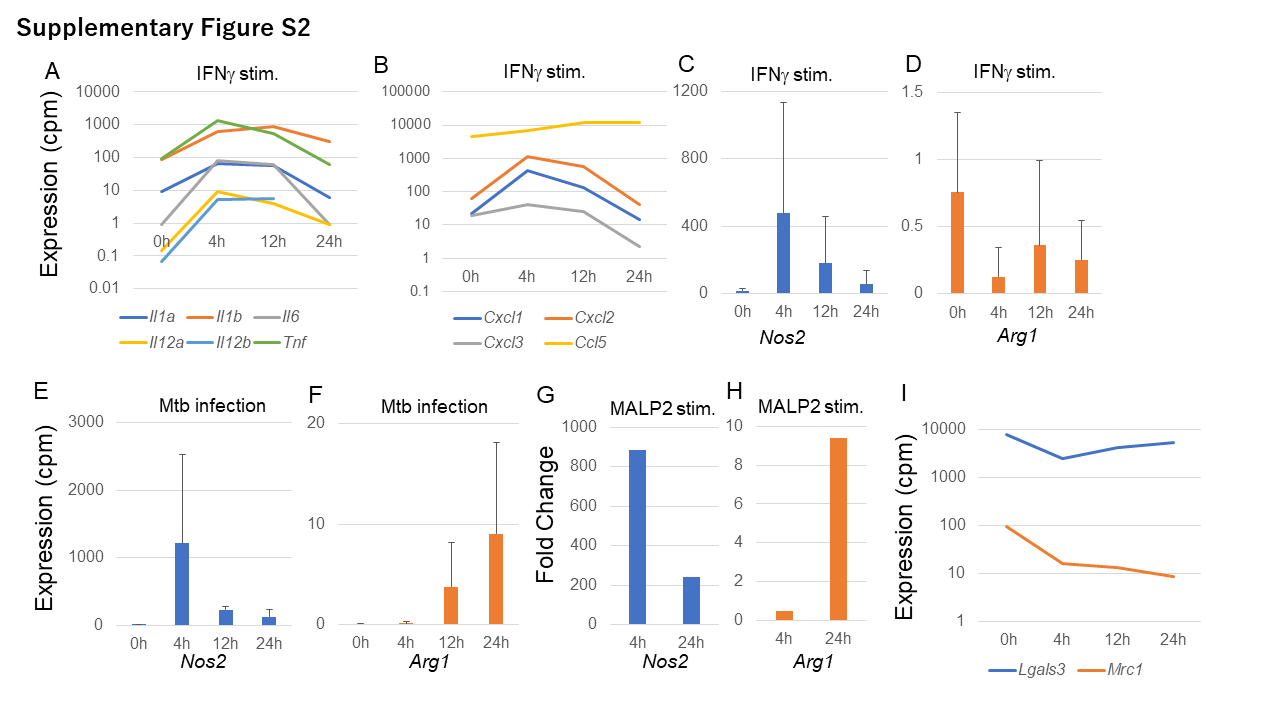

Supplement: Supplementary Figure 2 — Expressional change of M1-activated genes upon IFNγ stimulation, Mycobacterium tuberculosis (Mtb) infection, and MALP2 stimulation. Gene expression profiles for (A) cytokines, (B) chemokines, (C) Nos2, and (D) Arg1 in IFNγ-stimulated bone-marrow derived macrophages (BMDMs) and (E) of Nos2 and (F) Arg1 in Mtb-infected BMDMs. Average values of triplicate data are shown. The standard deviation is also shown in figures (C–F). The expression profiles were taken from the FANTOM5 database (40). The fold change of expression of Nos2 and Arg1 stimulated by the Tlr2 ligand MALP2 was calculated in comparison with no stimulation ((G, H), respectively) by RT-qPCR. Average results of duplicate experiments are shown. Expression profiles of two M2 marker genes, Lgals3 and Mrc1, in Mycobacterium avium complex-infected macrophages are also shown (I). Average expression values of quadruplicate data were plotted, but the standard deviation omitted. [file Image_2.tif]

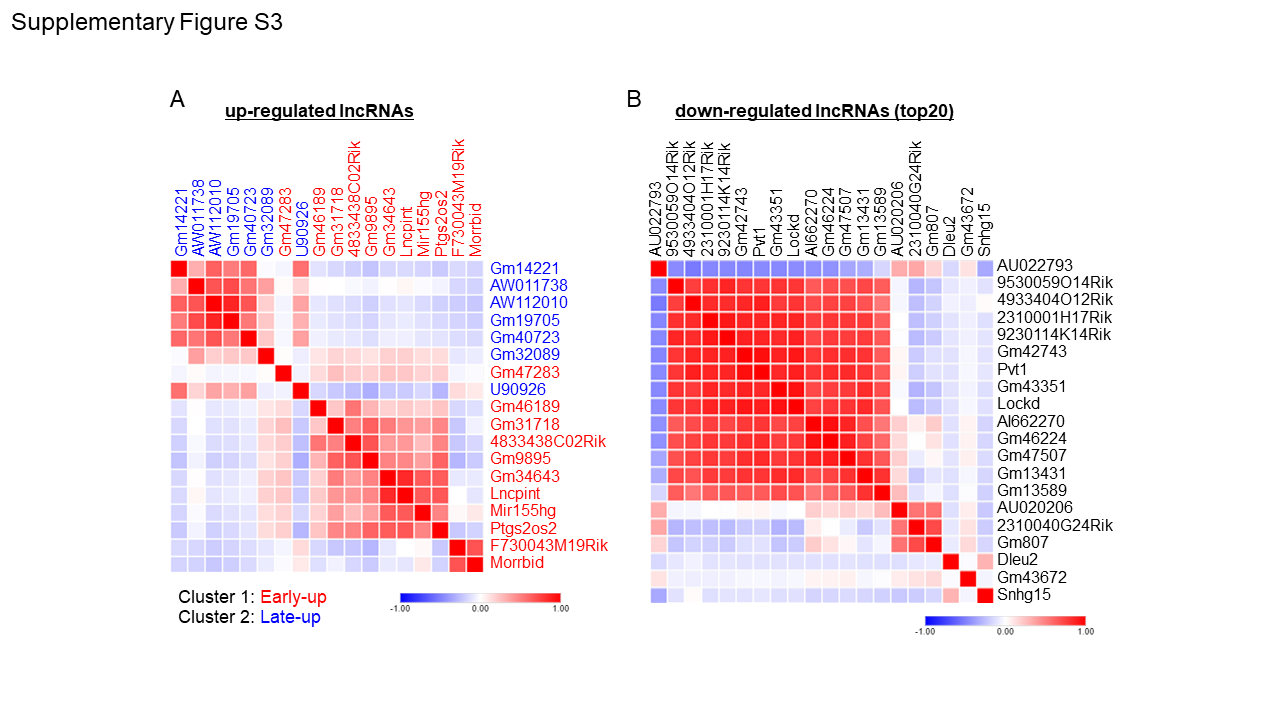

Supplement: Supplementary Figure 3 — Clustering analysis of predicted gene ontology (GO) biological process terms for long non-coding RNA (lncRNA)-associated protein-coding transcripts. We extracted protein-coding transcripts with Pearson correlation coefficients of both more than 0.8 and less than -0.8 with each differentially expressed lncRNA, and we then subjected them to GO analysis. Hierarchical clustering of the Pearson correlation coefficients of log-transformed P-values for GO biological process terms associated with (A) upregulated and (B) downregulated lncRNA-associated protein-coding transcripts. [file Image_3.tif]

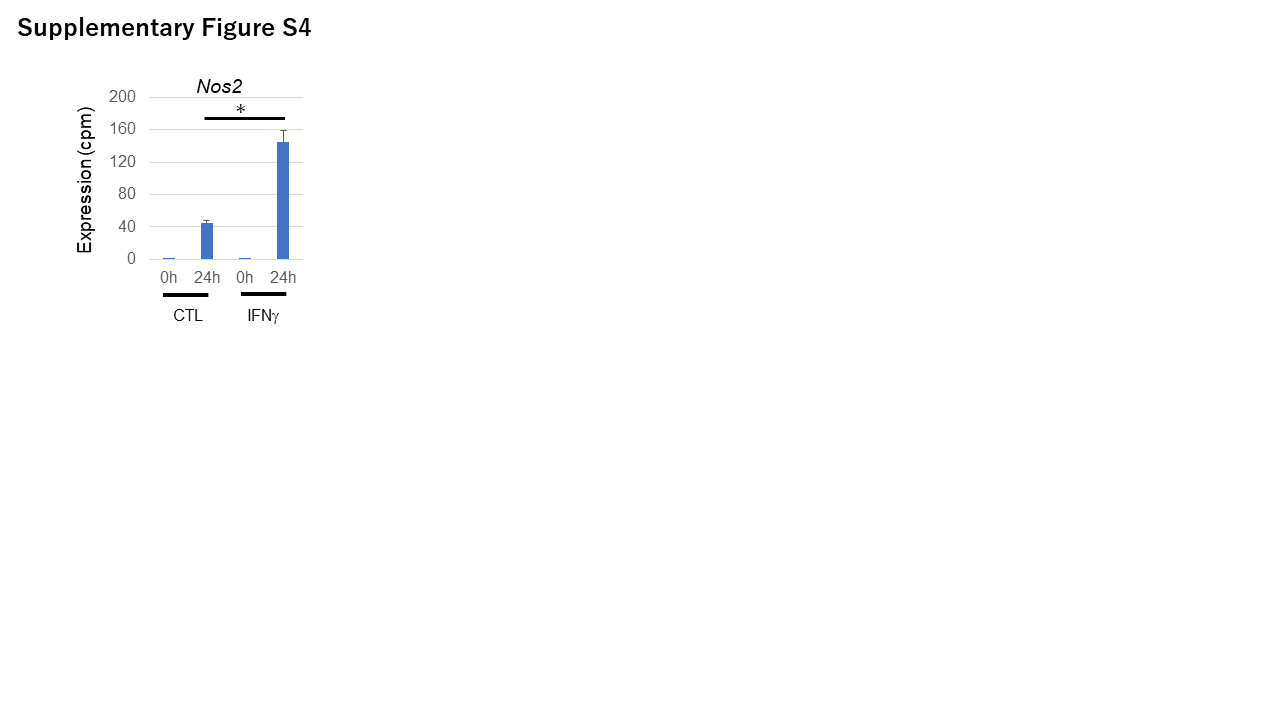

Supplement: Supplementary Figure 4 — Expressional change of Nos2 during Mycobacterium avium complex infection under no preactivation (CTL) or under M1 (IFNγ) preactivation. IFNγ stimulation was carried out 24 h prior to Mycobacterium avium complex (MAC) infection (M1 preactivation). After 0 and 24 h of the infection, total RNA was extracted from macrophages and subjected to CAGE-seq transcriptome analysis. Asterisks denote P value less than 0.01 with Student’s unpaired T test. [file Image_4.tif]

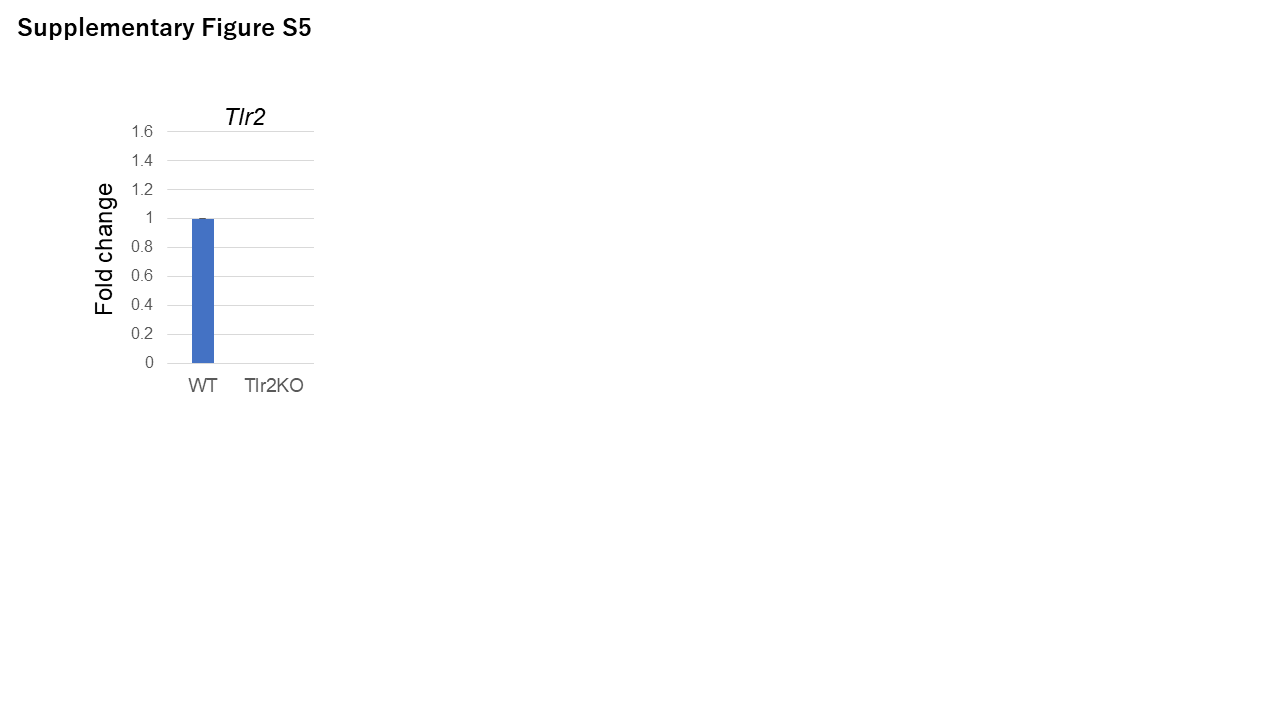

Supplement: Supplementary Figure 5 — Expression of Tlr2 genes. Tlr2 expression in wild-type (WT) and Tlr2 knockout (Tlr2KO) mouse bone-marrow derived macrophages (BMDMs) was measured by RT-qPCR to confirm that Tlr2KO BMDMs did not express Tlr2. [file Image_5.tif]

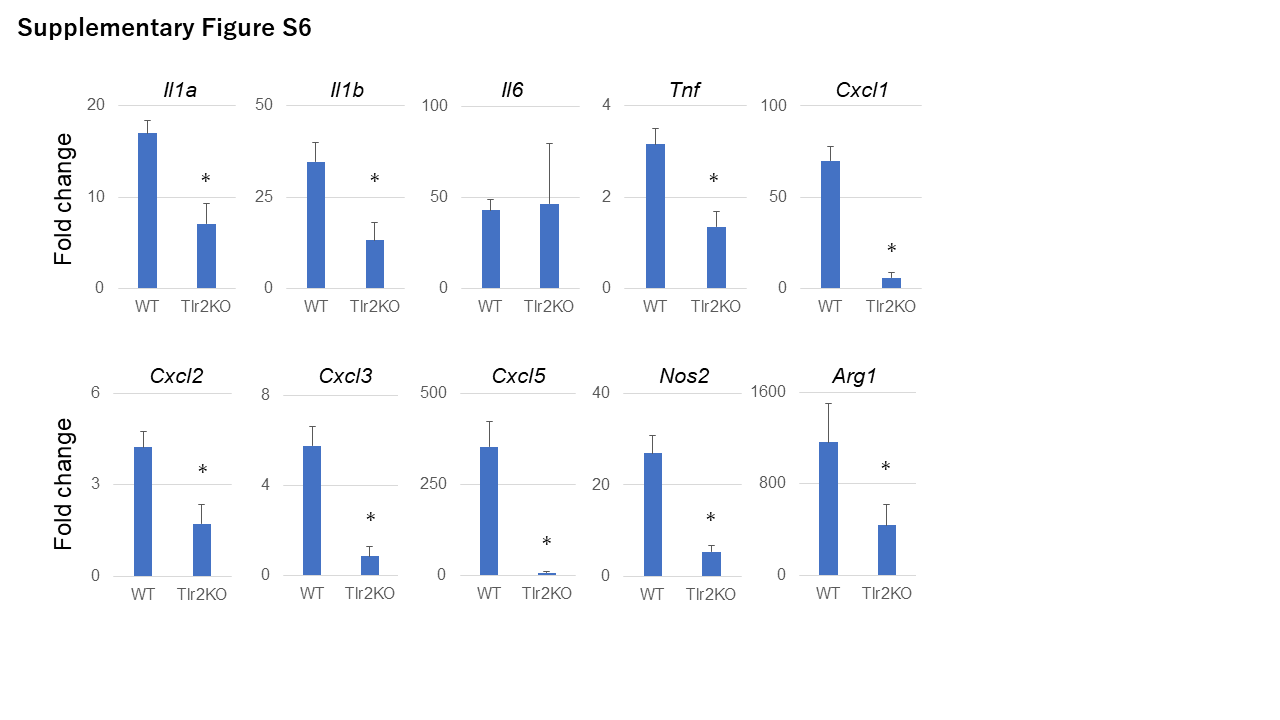

Supplement: Supplementary Figure 6 — Expressional fold change of several protein-coding genes in Mycobacterium avium complex-infected wild-type (WT) and Tlr2 knockout (Tlr2KO) macrophages by RT-qPCR. Expressional fold change for indicated genes was calculated between 0 and 24 h after Mycobacterium avium complex infection in WT and Tlr2KO macrophages by RT-qPCR. Asterisks denote P value less than 0.01 with Student’s unpaired T test between WT and Tlr2KO macrophages. [file Image_6.tif]

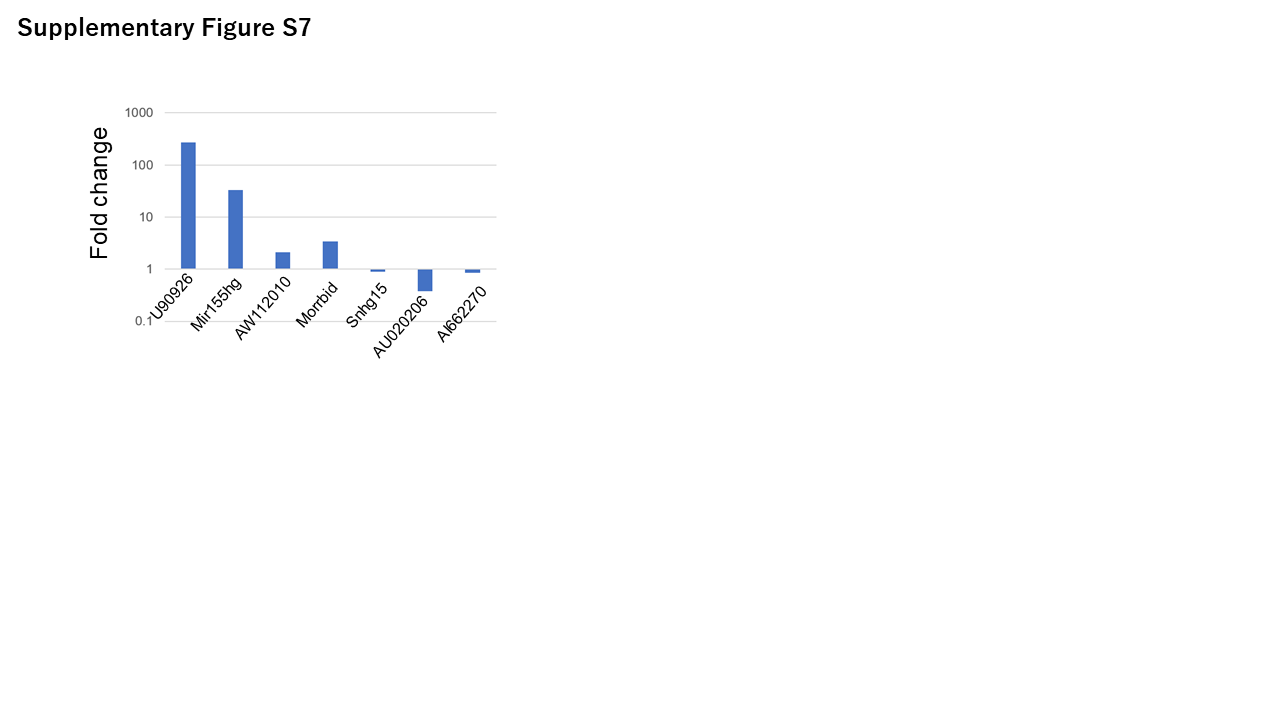

Supplement: Supplementary Figure 7 — Expressional fold change of representative long non-coding RNAs upon MALP2 stimulation. Expressional fold change for seven representative lncRNAs was calculated between 0 and 4 h after MALP2 stimulated macrophages by RT-qPCR. Average results of duplicate experiments are shown. [file Image_7.tif]
